# *MsHDZ23,* a Novel *Miscanthus HD-ZIP* Transcription Factor, Participates in Tolerance to Multiple Abiotic Stresses

**DOI:** 10.3390/ijms25063253

**Published:** 2024-03-13

**Authors:** Naixu Liu, Ruikang Yu, Wendi Deng, Ruibo Hu, Guo He, Kang He, Yingzhen Kong, Xianfeng Tang, Gongke Zhou, Congpeng Wang

**Affiliations:** 1 College of Resources and Environment, Qingdao Agricultural University, Qingdao 266109, China; liunaixu1029@163.com (N.L.);; 2 College of Landscape Architecture and Forestry, Qingdao Agricultural University, No. 700 Changcheng Road, Chengyang District, Qingdao 266109, China; dwder1119@163.com (W.D.); tangxf@qau.edu.cn (X.T.); 3 CAS Key Laboratory of Biofuels, Shandong Provincial Key Laboratory of Energy Genetics, Qingdao Institute of Bioenergy and Bioprocess Technology, Chinese Academy of Sciences, Qingdao 266101, China; hurb@qibebt.ac.cn (R.H.);; 4 Shandong Peanut Research Institute, Qingdao 266100, China; 5 College of Agronomy, Qingdao Agricultural University, Qingdao 266109, China; kongyzh@qau.edu.cn; 6 Academy of Dongying Efficient Agricultural Technology and Industry on Saline and Alkaline Land in Collaboration with Qingdao Agricultural University, Dongying 257000, China

**Keywords:** *Miscanthus sinensis*, *MsHDZ23*, drought, salt, alkali

## Abstract

The homeodomain-leucine zipper (HD-ZIP) transcription factors, representing one of the largest plant-specific superfamilies, play important roles in the response to various abiotic stresses. However, the functional roles of HD-ZIPs in abiotic stress tolerance and the underlying mechanisms remain relatively limited in *Miscanthus sinensis*. In this study, we isolated an HD-ZIP TF gene, *MsHDZ23*, from *Miscanthus* and ectopically expressed it in Arabidopsis. Transcriptome and promoter analyses revealed that *MsHDZ23* responded to salt, alkali, and drought treatments. The overexpression (OE) of MsHDZ23 in Arabidopsis conferred higher tolerance to salt and alkali stresses compared to wild-type (WT) plants. Moreover, *MsHDZ23* was able to restore the *hb7* mutant, the ortholog of *MsHDZ23* in Arabidopsis, to the WT phenotype. Furthermore, *MsHDZ23*-OE lines exhibited significantly enhanced drought stress tolerance, as evidenced by higher survival rates and lower water loss rates compared to WT. The improved drought tolerance may be attributed to the significantly smaller stomatal aperture in *MsHDZ23*-OE lines compared to WT. Furthermore, the accumulation of the malondialdehyde (MDA) under abiotic stresses was significantly decreased, accompanied by dramatically enhanced activities in several antioxidant enzymes, including superoxide dismutase (SOD), peroxidase (POD), and catalase (CAT) in the transgenic plants. Collectively, these results demonstrate that *MsHDZ23* functions as a multifunctional transcription factor in enhancing plant resistance to abiotic stresses.

## 1. Introduction

Unfavorable environmental factors, such as salt, alkali, and drought stress, severely limit the growth and development of sessile plants above ground. Plants have developed various coping strategies to survive in challenging environments [1]. They have evolved complex physiological and molecular mechanisms to withstand and maintain normal growth under stressful conditions. The activation of many stress-responsive genes leads to altered metabolic and physiological processes and is an important molecular mechanism for abiotic stress tolerance in plants [2]. Abscisic acid (ABA) is a well-known stress-related phytohormone that plays a key role in abiotic stress signaling cascades. Various genes associated with ABA-mediated stress signaling pathways have been identified in different plant species [3,4]. Drought, salt, or alkali stress also triggers the overproduction of reactive oxygen species (ROS), which can adversely affect cellular redox homeostasis and lead to oxidative stress [5,6]. In addition, transcription factors (TFs) have been recognized as key regulators in abiotic stress signaling networks, controlling the activation or repression of downstream stress-responsive genes [7,8]. TFs regulate abiotic stress signaling through pathways dependent or independent of ABA signaling [9]. The homeodomain-leucine zipper (HD-ZIP) TF family is of particular interest among the various TF families because of its crucial regulatory role in tolerating various abiotic stresses [10].

HD-ZIP proteins are plant-specific transcription factors that contain a homeodomain (HD) and a leucine zipper (LZ) domain. The highly conserved HD binds specifically to DNA, and the LZ mediates homodimer or heterodimer formation [11]. The HD-ZIP protein mediates stress tolerance in plants by regulating the expression of downstream stress-related genes mainly through the ABA-mediated signaling pathway [12]. In the model species *Arabidopsis*, the disruption of the HD-ZIP II genes *HAT1* and *HAT3* results in enhanced drought tolerance [13]. Notably, HAT1 directly binds to the promoters of the ABA biosynthesis genes *ABA3* and *NCED3* to negatively regulate their expression, resulting in reduced ABA accumulation [13]. Another HD-ZIP II protein, HAT22/ABIG1, showed increased expression in response to ABA and drought treatments and shared a similar regulatory pattern with two other known ABA signaling genes, *PYL6* and *CIPK12*, suggesting that HAT22/ABIG1 may be an important component of the ABA signaling pathway [14]. In Miscanthus, the HD-ZIP TF *MsHB7* negatively regulates salt tolerance by inhibiting the expression of the ABA-response genes *MsSnRK2.6* and *MsABI1* [15]. Similarly, HD-ZIP TF CaHDZ12 in chickpeas plays a role in ABA-dependent signaling and contributes to abiotic stress tolerance. It promotes osmolyte production, reduces intracellular ROS levels, and induces the expression of *SnRK2* kinase and other stress-related genes in response to abiotic stresses [16]. Additionally, HD-ZIP proteins have been identified in eucalyptus [17], wheat [18,19,20], maize [21,22,23], and rice [24,25,26,27,28], where they play diverse roles in abiotic stress responses by controlling plant development and influencing stress-related gene expression.

Despite great progress in stress-related HD-ZIP proteins in model plants and crops, little is known about HD-ZIP genes in abiotic stresses in *Miscanthus*. *Miscanthus* is a perennial rhizomatous C4 grass native to East Asia [29]. *Miscanthus* has outstanding characteristics, such as having a high biomass yield, being rich in polysaccharides, and having a wide adaptability to various environments (especially marginal lands) and low water and fertilizer requirements [30], making it a promising lignocellulosic bioenergy crop in China [31,32,33]. The cultivation of *Miscanthus* on marginal land (e.g., saline soil) that is not arable for agronomic crops will not only provide sufficient raw materials for second-generation bioethanol production, but will also aid in soil reclamation, CO2 emission reduction, and carbon sequestration and neutralization [34]. However, compared with the main crops, the genetic improvement of *Miscanthus* lags far behind because of its complex genetic background and the unclarified molecular mechanisms underlying agronomic traits and abiotic stress tolerance [35]. In the present study, we identified 48 HD-ZIP proteins from *M. sinensis*. Among these HD-ZIP proteins, *MsHDZ23* is dramatically induced under alkali, mixed salt–alkali, and prolonged drought stresses. We carried out a detailed functional characterization of *MsHDZ23* in abiotic stress responses in transgenic Arabidopsis. Our results demonstrate that *MsHDZ23* plays a positive role in drought, alkali, and salinity stress tolerance. The enhanced tolerance of transgenic plants was achieved via the activation of antioxidant enzymes in ROS scavenging. The results lay the theoretical foundations for the further improvement of *Miscanthus* stress tolerance by means of genetic engineering.

## 2. Results

### 2.1. Genome-Wide Identification of HD-ZIP Genes in M. sinensis

The HD-ZIP protein sequences were identified via a local BLAST search against the annotated genome of *M. sinensis* using the HMM profile of the HD and LZ domain. The presence of the HD and LZ domain was further confirmed with a conserved domain (CD) search at Phytozome. Ultimately, 48 *HD-ZIP* genes were identified in the *M. sinensis* genome. They were designated *MsHDZ1* to *MsHDZ48*, based on the physical locations on chromosomes. Like the *Arabidopsis HD-ZIP* family, the 48 *HD-ZIP* transcription factor genes of *M. sinensis* were divided into four subfamilies (Figure S1). Detailed information on *M. sinensis HD-ZIP* genes and their closest *Arabidopsis* orthologs is provided in Supplementary Table S1. Generally, there were one or two *MsHDZ* orthologs for each *Arabidopsis HDZ* gene.

### 2.2. Expression Profiling of MsHD-ZIP Genes in Multiple Abiotic Stresses

To explore the expression profile of *MsHD-ZIP* genes in response to drought, salt, or/and alkaline stress, we mined their expression profiles using the transcriptome data (unpublished data). At least 18, 12, 13, 15, and 14 *MsHD-ZIP* genes exhibited significantly upregulated expression (more than two-fold change) under short drought, long drought, salt, alkali, and mixed salt–alkali stresses, respectively (Figure S2). For example, the expression of *MsHDZ17*, *MsHDZ34*, *MsHDZ37*, and *MsHDZ42* was specifically upregulated under salt stress, while the expression of *MsHDZ22* was specifically upregulated under salt and prolonged drought stresses. Moreover, the expression of *MsHDZ4*, *MsHDZ10*, *MsHDZ25*, and *MsHDZ31* was upregulated under mixed salt–alkali and prolonged drought stresses. Significantly, the expression of *MsHDZ23*, which responds to various stresses, was upregulated under alkali, mixed salt–alkali, and prolonged drought stresses, highlights its function in the stress resistance of *M. sinensis*.

### 2.3. MsHDZ23 Was Induced by Multiple Abiotic Stresses

**Figure 1:**
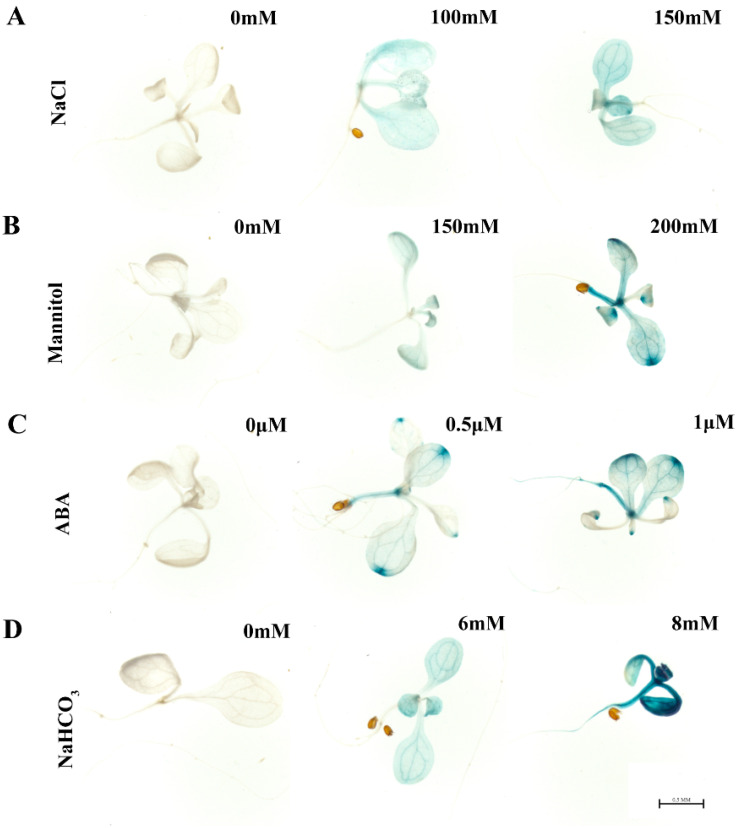
GUS expression in seedlings of Arabidopsis under various treatments. Transgenic Col-0 harboring *MsHDZ23 promoter::GUS* (the *GUS* reporter gene fused with the promoter sequence of −2500 bp from the first ATG) were analyzed after exposure to NaCl (**A**) (0, 100, and 150 mM), mannitol (**B**) (0, 150, and 200 mM), ABA (**C**) (0, 0.5, and 1 μM), and NaHCO_3_ (**D**) (0, 6, and 8 mM). The seedlings were grown on the normal 1/2 MS for 5 days and then moved to the treated medium for 7 days. Activation of the *MsHDZ23* promoter was observed via GUS staining (blue). It should be noted that MsHDZ23 mainly dyes the leaves. The scale bar is 0.5 mm.

To explore whether *MsHDZ23* responds to abiotic stress (Figure S2), we conducted a GUS staining assay using a *Pro_MsHDZ23_*::*GUS* construct in Miscanthus sinensis. The promoter region of MsHDZ23 (2500 bp) was cloned and fused with the GUS reporter gene. Germinated seeds were grown on 1/2 MS medium supplemented with different concentrations of ABA (0.5 μM, 1.0 μM), NaCl (100 mM, 150 mM), mannitol (150 mM, 200 mM), and NaHCO3 (5 mM, 7 mM) to induce abiotic stress. After 14 days, GUS staining was performed on the seedlings. Under different stresses, the leaves of seedlings showed GUS staining and the staining deepened with the increase in stress level (Figure 1). This result indicates that the expression of *MsHDZ23* is indeed induced by various abiotic stresses.

### 2.4. MsHDZ23 Is a Nucleus-Localized TF with Transactivation Activity

**Figure 2:**
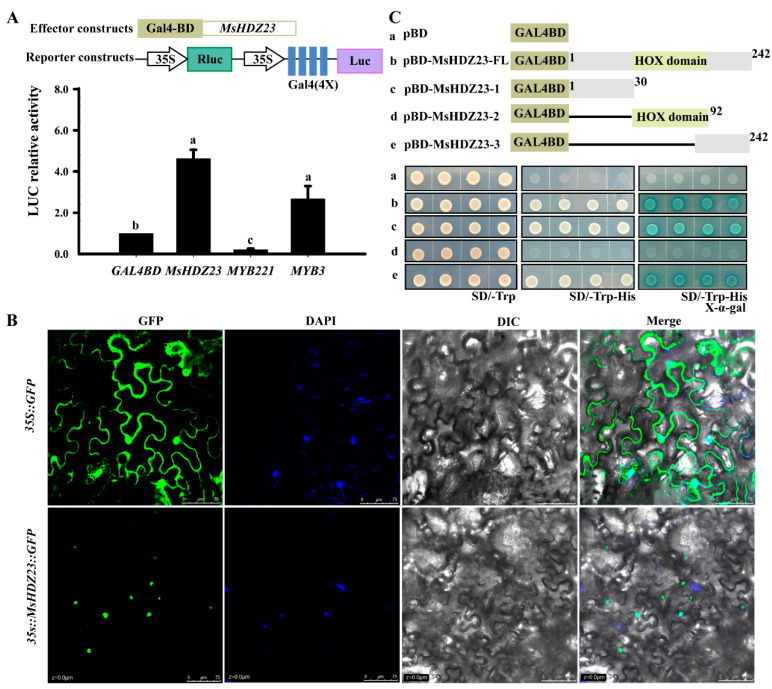
The MsHDZ23 protein has trans-activating activity and can be localized in the nucleus. (**A**) MsHDZ23 functions as a transcriptional activator. Arabidopsis leaf protoplasts were cotransfected with three reporter genes and one effector gene. The effector gene contains the yeast Gal4 BD fused in-frame with MsHDZ23, MYB221 (repressor control), or MYB3 (activator control). Data are the average of three independent experiments. (**B**) Subcellular localization of MsHDZ23. The recombinant vector pEarleyGate101-MsHDZ23-YFP and vector control were infiltrated into tobacco leaves separately. Fluorescence was observed under a laser scanning confocal microscope. DAPI staining indicates the location of cell nuclei. DIC: differential interference contrast. Bar = 100 μm. (**C**) Trans-activation activity assay of MsHDZ23 in yeast. Schematic diagram showing the structural domains in pGBKT7 of full-length (MsHDZ23-FL) and different truncated forms (N-terminal fragment, MsHDZ23-1, HOX structural domain, MsHDZ23-2, and C-terminal fragment, MsHDZ23-3) of MsHDZ23 fused to GAL4 DNA binding. The recombinant construct and pGBKT7 (negative control) were transformed into yeast strain AH109, respectively. The growth in the transformants was evaluated sequentially on SD/-Trp and SD/-Trp-His media. β-Galactosidase activity was assayed in the presence of X-α-gal substrate.

HD-ZIP proteins were predicated to be transcription factors in plants [10]. A transcriptional activation assay was performed using the Gal4 binding domain (BD)-MsHDZ23 fusion protein and a constitutively expressed reporter gene containing four upstream Gal4 DNA-binding sites (Gal4 [4X]-D1-3[4X]-GUS; Figure 2A). The data showed that *MsHDZ23* led to the activation of the reporter gene expression (Figure 2A). As controls, MYB3 and MYB221 were included to represent activation and inhibition controls, respectively, and their respective effects on the reporter gene expression were consistent with previous findings [36,37].

The transactivation activity assay of *MsHDZ23* was investigated using a yeast transactivation assay. Yeast cells containing the full-length sequence of CDS coding region (pBD-MsHDZ23-FL), the N-terminal domain (pBD-MsHDZ23-1), or the C-terminal domain (pBD-MsHDZ23-3) and vector control could grow on SD/−Trp medium, indicative of successful transformation. By contrast, transformants containing pBD-MsHDZ23-FL, pBD-MsHDZ23-1, or pBD-MsHDZ23-3 were able to grow on SD/−His medium (Figure 2B). Furthermore, yeast transformants containing these three constructs displayed strong β-galactosidase activity in the presence of X-α-gal (Figure 2B), indicating that *ADE2* and *HIS3* reporter genes were successfully activated by *MsHDZ23*.

The subcellular localization of *MsHDZ23* was examined via transient expression in the *Nicotiana. benthamiana* leaf epidermis. The GFP signals of the vector control were pervasively distributed throughout the cell without specific localizations. By contrast, the fluorescence signals of MsHDZ23-GFP were almost solely detected in the nuclei. This nuclear-specific localization was confirmed via co-staining with DAPI, which specifically labels DNA within the nucleus (Figure 2C).

### 2.5. MsHDZ23 Overexpression Affects the Development of Transgenic Arabidopsis

According to the phylogenetic tree (Figure S1) and Supplement Table S1, *MsHDZ23* and *Arabidopsis HOMEOBOX7* (*AtHB7*) are orthologous genes. To characterize the functional role of *MsHDZ23* in the plant stress response, we generated the overexpression lines of *MsHDZ23* (referred to as *MsHDZ23*-OE) and *hb7* mutant complement lines (referred to as *MsHDZ23*-complemented *hb7*) in *Arabidopsis*. The overexpression of MsHDZ23 had a noticeable impact on the morphogenesis characteristics of transgenic Arabidopsis, particularly on the leaves (Figure S3). The rosette leaves of the MsHDZ23-OE lines exhibited a distinct phenotype, with shortened lengths and longer petioles, which is in striking contrast to the typically long and narrow shape of the WT and *hb7* leaves (Figure S3A,B,D). Accordingly, the leaf size was substantially reduced in the MsHDZ23-OE lines compared to WT (Figure S3A,B).

### 2.6. MsHDZ23 Overexpression Improves Salt Tolerance

**Figure 3:**
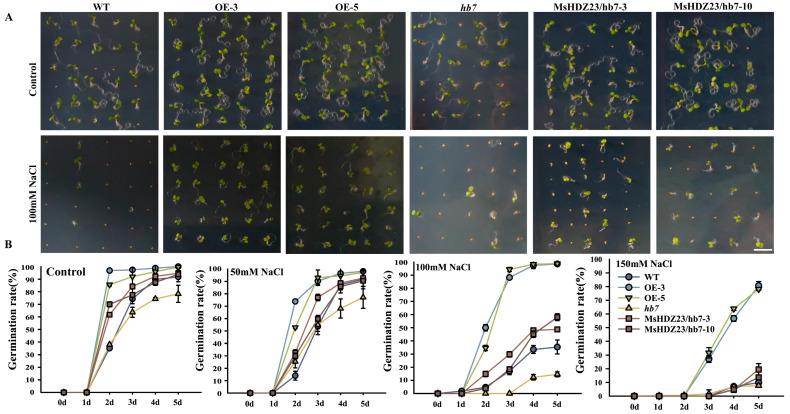
Overexpression of *MsHDZ23* promotes seed germination under NaCl stress. (**A**) Germination assay under control and 100 mM NaCl treatment. Bar: 0.5 cm. (**B**) Quantification of greening cotyledon frequency under control, 50 mM, 100 mM, and 150 mM NaCl treatment. The greening cotyledon rate was calculated as the percentage of seedlings with green cotyledon out of the whole seedlings. Data represent the mean of three replicates with 40 seeds for each genotype. Error bars represent standard deviation.

The salt tolerance of *MsHDZ23*-OE lines was investigated by measuring germination rates under salt (NaCl) treatments (Figure 3). In the control 1/2 MS medium, the germination rate of both the MsHDZ23-OE lines and WT was over 90%, whereas only the *hb7* mutant had a lower germination rate of 78% (Figure 3B). However, there was no significant difference in germination rates between the *MsHDZ23*-complemented *hb7* lines and WT. Under50 mM NaCl treatment, the germination time of all genotypes was delayed by 1 day, but the germination rate was not significantly affected. Under treatment with 100 mM NaCl, the germination rates of WT, the *hb7* mutant, and the *MsHDZ23*-complemented *hb7* lines decreased to 35%, 14.5%, 48.6%, and 58%, respectively, while the *MsHDZ23*-OE lines showed only a slight reduction in germination rate, accompanied by delayed germination (Figure 3B). Additionally, the *MsHDZ23*-OE lines displayed very vigorous growth characteristics, while a substantial inhibitory effect was observed for the WT seedlings (Figure 3A). When exposed to 150  mM NaCl, the difference in seed germination was dramatically increased between the overexpression lines and other genotypes. The germination rate of the *MsHDZ23*-OE lines was about 8 times higher than that of WT (Figure 3B).

The response of *MsHDZ23* overexpression to salt stress was further assessed via root elongation assays (Figure S3). All genotypes exhibited an equivalent root length when vertically cultivated under normal conditions (Figure S3A). However, significant differences were observed between all genotypes when exposed to 50 mM, 100  mM, or 150  mM NaCl treatment (Figure S3B). The primary root of the *MsHDZ23*-OE lines was dramatically longer compared to the other genotypes. Under three different concentrations of salt stresses, the *MsHDZ23*-OE lines were 55–57%, 35–38%, and 35–41% longer than WT, respectively. The *hb7* mutant exhibited the shortest root length under 100 mM NaCl salt stress, while the *MsHDZ23*-complemented *hb7* lines displayed a root length 25% longer than WT.

**Figure 4:**
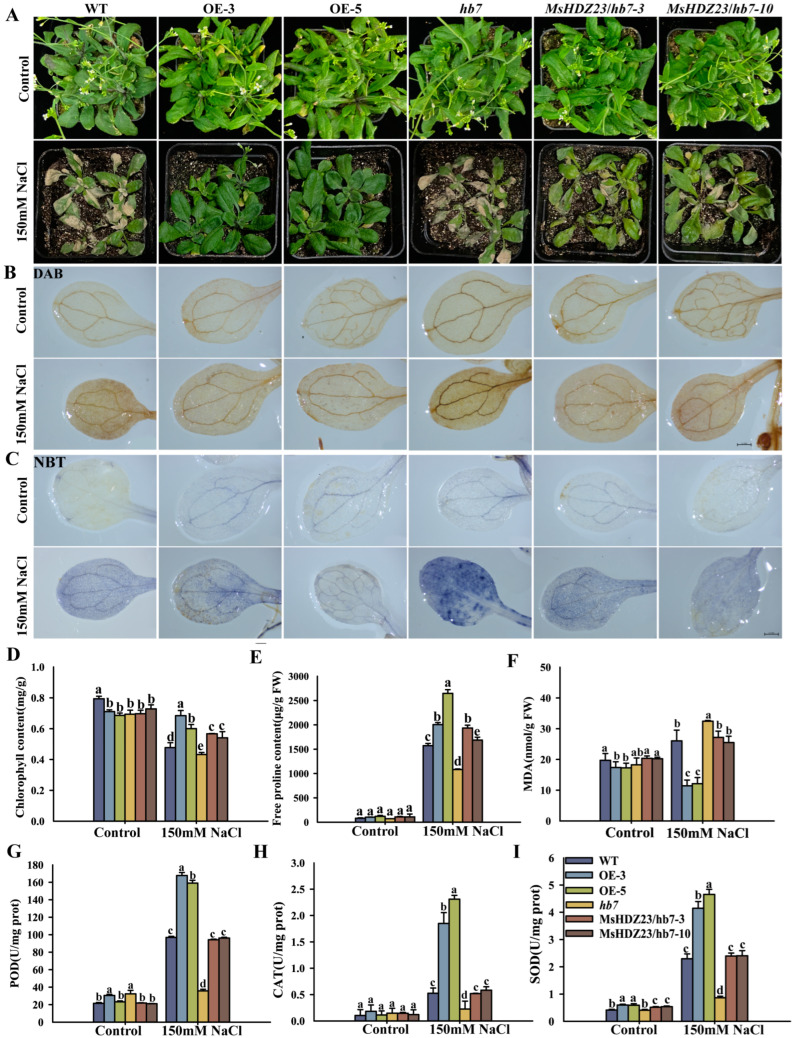
In transgenic Arabidopsis, overexpression of *MsHDZ23* improves salt stress tolerance. (**A**) Performance of *MsHDZ23* transgenesis lines, *hb7* mutant, and WT plants under salt stress. Three-week-old plants were adequately irrigated with NaCl solution (150 mM) for 12 d. (**B**,**C**) Histochemical assays to detect the accumulation of H_2_O_2_ and O^•2−^ via DAB (**B**) and NBT (**C**) staining under control and NaCl conditions. The scale bar is 0.5 mm. (**D**,**E**) Chlorophyll (**D**) and free proline (**E**) content of *MsHDZ23* transgenesis lines, *hb7* mutant and WT plants under NaCl treatments. (**F**–**I**) Quantification of MDA content and anti-oxidant enzyme activities. The MDA (**F**) level and enzymatic activities of SOD (**G**), POD (**H**), and CAT (**I**) were measured after 150 mM NaCl treatment for 12 d. Values are averages of three independent measurements. Error bars represent standard deviation. The different letters represent significant differences between lines (*p* ≤ 0.05).

We further examined the performance of the *MsHDZ23*-OE lines under salt stress during the vegetative growth stage (Figure 4). Upon NaCl treatment, most of the *hb7* mutant were severely wilted and eventually perished compared to WT (Figure 4A). In contrast, the majority of the *MsHDZ23*-OE lines maintained a healthy green appearance, with only slight leaf wilting observed (Figure 4A). Under the control conditions, the chlorophyll content of the *MsHDZ23*-OE lines was 10–13% lower than that of WT. However, under 150 mM salt stress, the chlorophyll content of the OE lines was 26% and 43% higher than that of WT, respectively (Figure 4D). Similarly, the chlorophyll content of the *MsHDZ23*-complemented *hb7* lines was 25–31% higher than that of the *hb7* mutant. The proline content, an essential index of plant stress resistance, was also evaluated. The results revealed that under salt stress, the proline content of the *MsHDZ23*-OE lines was 27–68% higher than that of WT. The MsHDZ23-complemented hb7 lines showed an even higher proline content, with values 56% to 79% higher than that of the hb7 mutant and even exceeding that of WT (Figure 4E).

To ascertain whether the ROS level is altered in transgenic and mutant lines, we performed in situ histochemical assays of ROS accumulation via DAB and NBT staining. Under normal conditions, the colors developed via DAB and NBT staining in all the lines were relatively light, and there were no significant differences in ROS levels between the transgenic lines, mutant lines, and WT (Figure 4B,C). When exposed to salt treatment, the coloration of DAB and NBT staining was drastically deepened in the leaves of all genotypes. However, the leaves of the MsHDZ23-OE lines exhibited a lighter staining color compared to the other genotypes, with the mutants displaying the darkest color, suggesting that the overexpression of *MsHDZ23* alleviates ROS generation under abiotic stress (Figure 4B,C). As an indicator of oxidative damage, the content of MDA generated during the peroxidation of membrane lipids is commonly used under abiotic stresses [38,39]. Equivalent amounts of MDA were detected in all genotypes under normal conditions. However, when exposed to salt stress treatment, only 44–46% levels of MDA were detected in the *MsHDZ23*-OE lines compared to WT, and the *MsHDZ23*-complemented hb7 lines showed MDA levels similar to WT (Figure 4F).

Since the formation of ROS in the cells can be promoted by most abiotic stresses and subsequently hurts the plants [40,41], we postulate that the overexpression of *MsHDZ23* can probably inhibit the production of ROS and/or facilitate the removal of the oxidative products to protect plants from membrane damage. To validate this hypothesis, we determined the activities of CAT, POD, and SOD before and after stress treatments. Under normal conditions, the enzyme activities were comparable in all genotypes. Under salt stress conditions, enzyme activities increased in all genotypes. However, more dramatic increases were observed for the *MsHDZ23*-OE lines than for the other genotypes (Figure 4G–I). The most obvious difference was observed in CAT activity. Under stress conditions, the activity of CAT enzyme in *MsHDZ23*-OE lines was 3.5–4.4 times higher than that of WT and 8–10 times higher than that of *hb7* mutant lines (Figure 4H). The enzyme activity of the *MsHDZ23*-complemented *hb7* lines was similar to that of WT. These data suggest that *MsHDZ23* overexpression stimulates the activities of anti-oxidative enzymes and enhances the ROS-scavenging capability. Collectively, these results indicate that *MsHDZ23*-OE lines have a superior salt stress tolerance compared with WT, and *MsHDZ23* can restore the tolerance of *hb7* mutants to that of WT.

### 2.7. MsHDZ23 Overexpression Improves Alkali Tolerance

**Figure 5:**
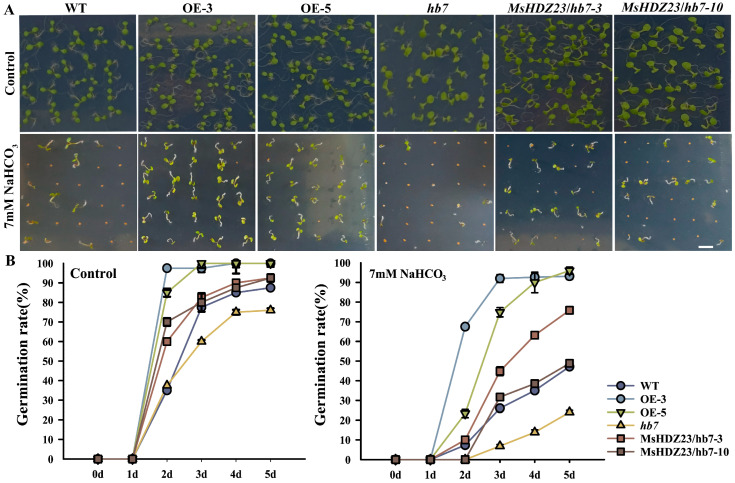
Overexpression of *MsHDZ23* promotes seed germination under NaHCO_3_ stress. (**A**) Germination assay under control and 7 mM NaHCO_3_ treatment. Bar: 0.5 cm. (**B**) Quantification of greening cotyledon frequency under control and 7 mM NaHCO_3_ treatment. The greening cotyledon rate was calculated as the percentage of seedlings with green cotyledon out of the whole seedlings. Data represent the mean of three replicates with 40 seeds for each genotype. Error bars represent standard deviation.

In order to assess the function of *MsHDZ23* in alkali stress, the germination rate of each genotype under 7 mM NaHCO_3_ stress was determined (Figure 5). Treatment with 7 mM NaHCO_3_ significantly decreased the germination rate of WT, *MsHDZ23*-complemented *hb7* lines, and *hb7* mutant. However, in the *MsHDZ23*-OE lines, although there was a delay in germination, the germination rate was only slightly reduced (Figure 5). Under 7 mM NaHCO_3_ stress, the germination rate of *MsHDZ23*-OE lines was 95–100% higher than that of WT. These results indicate that the overexpression of *MsHDZ23* enhances the tolerance to NaHCO_3_ to promote seed germination under alkali stress conditions.

**Figure 6:**
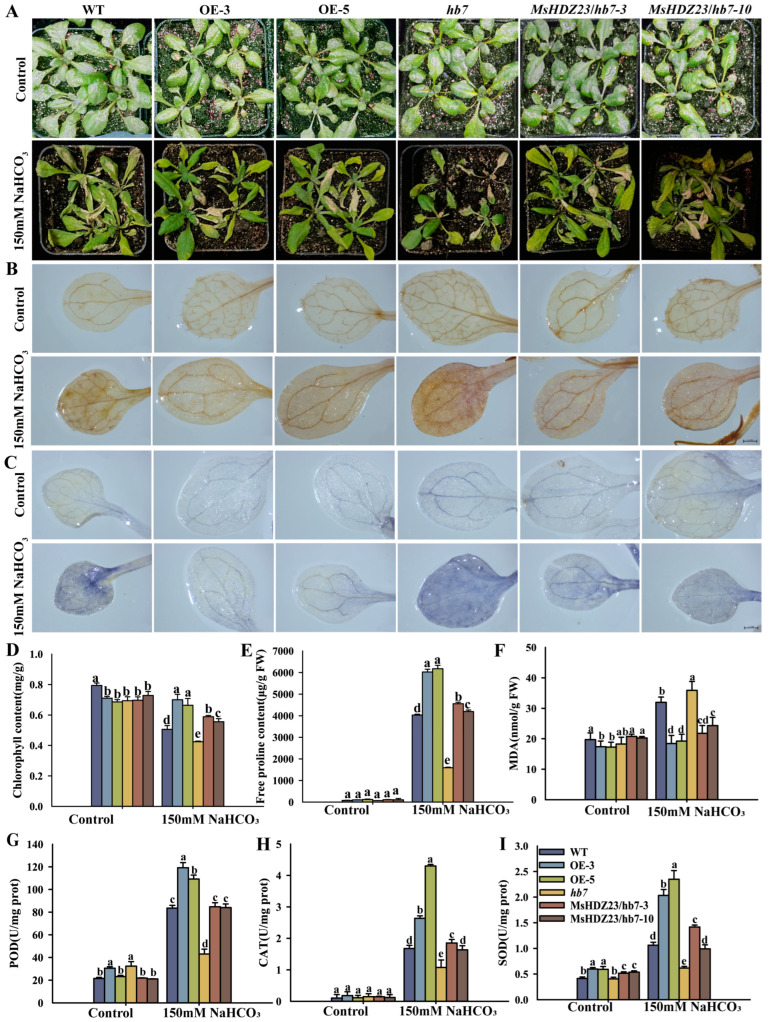
Overexpression of *MsHDZ23* improves alkali stress tolerance in transgenic Arabidopsis. (**A**) Performance of *MsHDZ23* transgenesis lines, *hb7* mutant lines, and WT plants under alkali stress. Three-week-old plants were adequately irrigated with NaHCO_3_ solution (150 mM) for 12 d. (**B**,**C**) Histochemical assays to detect the accumulation of H_2_O_2_ and O^•2−^ via DAB (**B**) and NBT (**C**) staining under control and NaHCO_3_ conditions. The scale bar is 0.5 mm. (**D**,**E**) Chlorophyll (**D**) and free proline (**E**) content of *MsHDZ23* transgenesis lines, *hb7* mutant lines, and WT plants under NaHCO_3_ treatments. (**F**–**I**) Quantification of MDA content and anti-oxidant enzyme activities. The MDA (**F**) level and enzymatic activities of SOD (**G**), POD (**H**), and CAT (**I**) were measured after 150 mM NaHCO_3_ treatment for 12 d. Values are averages of three independent measurements. Error bars represent standard deviation. The different letters represent significant differences between lines (*p* ≤ 0.05).

We further investigated the tolerance of the *MsHDZ23*-OE lines, *hb7* mutant, *MsHDZ23*-complemented *hb7* lines, and WT to NaHCO_3_ stress at the early vegetative stage. The three-week-old soil-grown plants were irrigated with 125 mM NaHCO_3_ every 3 days for a period of 2 weeks. The results of the survey showed that the *MsHDZ23*-OE lines demonstrated better stress tolerance to NaHCO_3_ than the other genotypes (Figure 6A). Under normal condition, almost no difference was found in plant growth, chlorophyll contents, proline contents, or MDA levels among all genotypes (Figure 6D–F). However, in the present of 125 mM NaHCO_3_, the chlorophyll content in WT was decreased by 36.7%, the *hb7* mutant was decreased by 40%, and the two *MsHDZ23*-OE lines only exhibited a decrease of 1.4% and 2.9% (Figure 6D). The change trend in proline was opposite to that of chlorophyll under NaHCO_3_ stress. Conversely, the proline content increased in the MsHDZ23-OE lines under NaHCO3 stress compared to that of WT, with an increase of 49% and 53%, while the proline content in the mutant was the lowest, only 39% of that in WT. The MSHDZ23-complemented hb7 line showed proline content levels similar to WT (Figure 6E). Moreover, the MDA levels demonstrated that MsHDZ23 reduced the damage caused by high alkaline stress in plants (Figure 6F). The results of DAB and NBT staining showed that the accumulation of ROS in the *MsHDZ23*-OE lines under alkali stress was the least among all genotypes, which was consistent with the results for salt stress (Figure 6B,C). Furthermore, the activities of CAT, POD, and SOD in the *MsHDZ23*-OE lines treated with NaHCO_3_ were higher than those in the other genotypes (Figure 6G–I). This suggests that *MsHDZ23*-OE lines are more efficient in removing ROS products and additional free radicals to protect cell structures from damage.

### 2.8. Overexpression of MsHDZ23 Enhances Drought Tolerance

**Figure 7:**
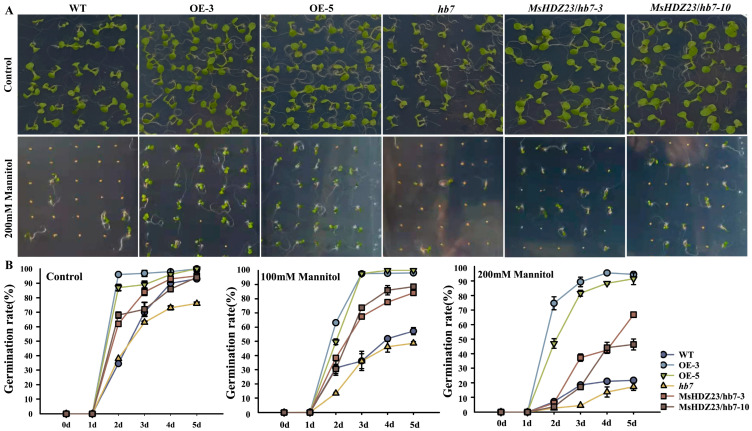
Overexpression of *MsHDZ23* promotes seed germination under mannitol stress. (**A**) Germination assay under control and 200 mM mannitol treatment. (**B**) Quantification of greening cotyledon frequency under control, 100 mM, and 200 mM treatment. The greening cotyledon rate was calculated as the percentage of seedlings with green cotyledon out of the whole seedlings. Data represent the mean of three replicates with 40 seeds for each genotype. Error bars represent standard deviation.

Given the evidence of crosstalk between the signaling pathways that regulate different responses to salt, alkaline, and drought stresses [42,43], along with the response of *MsHDZ23* to prolonged drought in transcriptome analyses (Figure S1B), we investigated drought-tolerant phenotypes across the *MsHDZ23*-OE lines, *hb7* mutant lines, the *MsHDZ23*-complemented *hb7* lines, and WT plants. Under the treatment of 100 mM and 200 mM mannitol, the seeds of the *MsHDZ23*-OE lines exhibited a slower seed coat breakage pace and a slight reduction in germination rate (Figure 7). However, the germination rates of other genotypes decreased significantly with increasing stress intensity, particularly the *hb7* mutant and WT. The germination rates of the hb7 mutant decreased by 29.7% (100 mM) and 61% (200 mM), while the WT rates decreased by 34.6% (100 mM) and 70.1% (200 mM), respectively (Figure 7B). The results indicated that the overexpression of *MsHDZ23* enhanced tolerance to mannitol-induced osmotic stress.

**Figure 8:**
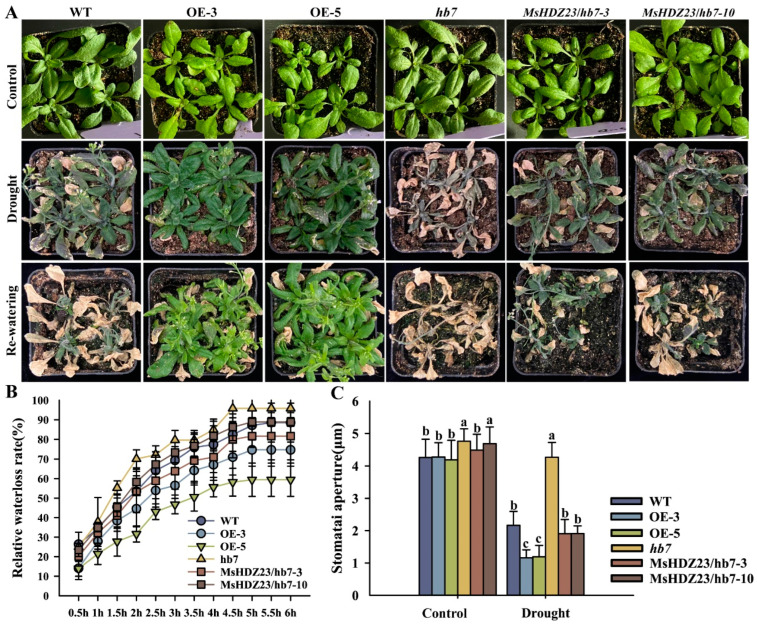
Overexpression of *MsHDZ23* improves drought stress tolerance in transgenic Arabidopsis. (**A**) Drought stress tolerance assay. The *MsHDZ23* transgenesis lines, *hb7* mutant lines, and WT plants were grown normally for four weeks in soil. Water supply was withheld for 12 d, then followed by re-watering for recovery. Images were taken 3 d after recovery. (**B**) Water loss rates of *MsHDZ23* transgenesis lines, *hb7* mutant, and WT plant leaves. (**C**) Stomatal aperture of rosette leaves of MsHDZ23 transgenesis lines, *hb7* mutant and WT plants under drought stress tolerance. Values are averages of three independent measurements. Error bars represent standard deviation. The different letters represent significant differences between lines (*p* ≤ 0.05).

The drought stress tolerance of the *MsHDZ23*-OE lines was further assessed at the vegetation growth stage (Figure 8A). After the dehydration stress, WT, *hb7* mutant, and *MsHDZ23*-complemented *hb7* lines displayed extreme leaf wilting due to a severe water deficiency. However, the *MsHDZ23*-OE lines only exhibited a slight wilting phenotype, with most of the leaves remaining turgid and maintaining a healthy green color (Figure 8A). After 3 d of resuming watering, only a few of WT plants survived and all the *hb7* mutants died. In contrast, most of the *MsHDZ23*-OE lines resumed growth (Figure 8A). During the six-hour dehydration stress of excised leaves, a steady weight loss resulting from leaf evaporation was observed in all genotypes. However, a much lower water loss rate was always observed for the *MsHDZ23*-OE lines compared to other genotypes at all designated time points (Figure 8B). Stomatal movement is one of the main physiological responses of plants to drought stress. Under normal conditions, the stomata of all genotypes were open and the pore size of mutants was the largest. Under drought stress, the stomata of the *MsHDZ23*-OE lines were closed, while those of mutants remained open (Figure 8C). The results showed that *MsHDZ23* could enhance the sensitivity of stomatal opening and closing to drought stress, thereby controlling the movement and changes in stomatal size and ultimately improving plant drought resistance.

The results of NBT and DAB staining showed that the *MsHDZ23*-OE lines had the lightest staining, while the *hb7* mutant had the deepest staining, which was similar to the results for salt and alkali treatment (Figure S5A,B). The MDA content analysis showed that after drought treatment, the MDA content of the *MsHDZ23*-OE lines was 62.9% and 60.7% of WT (Figure S5C). Even after re-watering, the MDA content of WT was 24.9% and 40.4% higher than that of the *MsHDZ23*-OE lines and did not recover to control level. The CAT enzyme activity data showed that the *MsHDZ23*-OE lines had the highest enzyme activity after drought, which was 1.5 and 2.5 times higher than WT. In contrast, the hb7 mutant displayed the lowest enzyme activity, with values of only 25.7% and 15.7% of the OE lines, aligning with the results obtained for salt and alkali stress (Figure S5E). After re-watering, the CAT enzyme activity of the OE lines was still higher than that of other genotypes and did not return to a growth state consistent with the control. Similar trends were observed for SOD and POD enzyme activities (Figure S5D,F). These results suggest that *MsHDZ23* stimulates anti-oxidant enzymes and enhances ROS-scavenging capability under drought stress.

### 2.9. MsHDZ23 Overexpression Confers ABA Insensitivity

**Figure 9:**
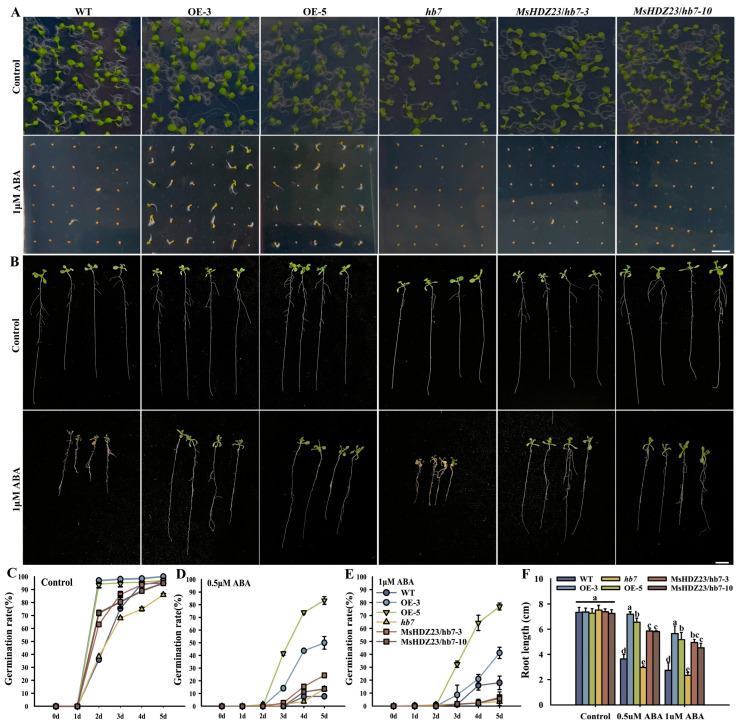
*MsHDZ23* overexpression confers ABA insensitivity in germination and root elongation. (**A**) Germination assay of *MsHDZ23* transgenesis lines, *hb7* mutant lines, and WT plants under control and 1 μM ABA treatment. (**C**–**E**) Quantification of greening cotyledon frequency under control, 0.5 μM, and 1 μM treatment. The greening cotyledon rate was calculated as the percentage of seedlings with green cotyledon out of the whole seedlings. Data represent the mean of three replicates with 40 seeds for each genotype. Error bars represent standard deviation. (**B**) Root elongation assay under ABA treatment. Images were taken 7 d after vertical cultivation. The scale bar is 1 cm. (**F**) Quantification of primary root length under different concentrations of ABA treatment (0, 0.5, 1 μM) for 5 d. Data represent the mean of three replicates with 20 plants for each genotype. Error bars indicate the standard deviation. The different letters represent significant differences between lines (*p* ≤ 0.05).

The GUS staining of *Pro_MsHDZ23_:: GUS* lines showed that *MsHDZ23* was induced by the ABA signal (Figure 1C). We examined the sensitivities of the *MsHDZ23*-OE lines, WT, *hb7* mutant lines, and *MsHDZ23*-complemented *hb7* lines to exogenous ABA (Figure 9A). When grown on 1/2 MS supplemented with 0.5  μM or 1.0  μM ABA, there were dramatic reductions in germination rates in all genotypes (Figure 9A,C–E). On 0.5 μM ABA medium, the germination rate of the *MsHDZ23*-OE lines was 6.4 times and 10.7 times that of WT (Figure 9C). This indicated that the inhibitory effect of exogenous ABA on seed germination was alleviated in the *MsHDZ23*-OE lines (Figure 9A,C–E).

A root elongation assay was further performed under ABA treatment. The exogenous application of 0.5 or 1.0  μM ABA significantly inhibited root elongation for all seedlings (Figure 9B). However, the hb7 mutant exhibited more pronounced reductions in root length, followed by WT, while the MsHDZ23-OE lines were the least affected. This indicates that *MsHDZ23* reduces the sensitivity to ABA (Figure 9F).

## 3. Discussion

Saline, alkaline, and drought stresses represent the main environmental factors that limit crop growth and reduce crop productivity worldwide [44]. Understanding the mechanisms of plant responses to alkaline, saline, or drought stress and identifying resistant genes are crucial to developing biotechnological strategies to improve crop resistance [45]. *Miscanthus sinensis*, a forage, ornamental, and energy crop known for its high biomass, photosynthesis potential, and water use efficiency, can withstand various abiotic stresses and is widely cultivated all over the world [46]. The recent publication of the chromosome-scale assembly of the *M. sinensis* genome has provided a precious genetic resource for the improvement of Miscanthus breeding and the mining of a stress-resistant molecular regulatory network [47].

A couple of transcription factors were found to be inducible in the transcriptome profiles of *M. sinensis* under multiple abiotic stresses. Among these transcription factors, the HD-ZIP family is one of the prominently induced families. HD-ZIP proteins comprise a large family of plant-specific TFs, which plays a crucial role in the growth and development of plants and stress responses [10]. Transcriptome analysis revealed diverse expression patterns of HD-ZIP genes in response to abiotic stresses (Figure S1). Through bioinformatic analysis, we identified 48 *HD-ZIP* TFs in *Miscanthus* (Supplement Table S1), 35 of which responded to stresses (Figure S1), whereas no HD-ZIP proteins from *M. sinensis* have been verified, and their physiological and biological functions have remained unknown until now. Among these transcription factors, *MsHDZ23* has an obvious response to alkali, saline–alkali mixture, and long-term drought stresses (Figure S1). We carried out a detailed functional characterization of *MsHDZ23* in response to abiotic stresses. As *Miscanthus* is recalcitrant to be transformed and, currently, the transformation platform has not been established, we had to resort to studying the function of *MsHDZ23* in Arabidopsis. Our results demonstrated that *MsHDZ23* functions as a stress-responsive HD-ZIP activation factor and plays a critical role in saline, alkaline, and drought stress tolerance in transgenic Arabidopsis. *MsHDZ23* is localized in the nucleus and shows transactivation activity (Figure 2), which is in accordance with the typical characteristics of TFs.

HD-ZIP TFs have been revealed to mediate the abiotic stress response through ABA-dependent signaling pathways [13,15,48]. There is compelling evidence supporting the role of *MsHDZ23* in the abiotic stress response via an ABA-dependent pathway. Firstly, the expression of *MsHDZ23* was significantly upregulated when subjected to exogenous ABA (Figure 1). Secondly, the overexpression of *MsHDZ23* in Arabidopsis conferred decreased sensitivity to ABA, which was evidenced by the significantly higher seed germination and increased seedling root length in the overexpression lines compared to WT (Figure 9). The results indicate that the overexpression of *MsHDZ23* led to hyposensitivity to ABA, and enhanced drought, salt, and alkali stress tolerance (Figure 4, Figure 6 and Figure S4). Therefore, *MsHDZ23* may act as a positive transcriptional regulator in drought, salt, and alkali stresses tolerance in an ABA-dependent manner.

Abiotic stresses usually impose substantial oxidative or osmotic stress on plants [15]. As a result, massive amounts of ROS (e.g., H_2_O_2_ and O_2_^•−^) are generated. ROS, when present at a normal level, serve as important signaling molecules in various plant physiological processes. However, the excessive accumulation of ROS can lead to severe peroxidation damage to cellular membranes, resulting in the generation of massive secondary products, such as MDA [5,49]. To counterbalance the excessively accumulated ROS, plants have evolved intricate defensive strategies, among which various types of antioxidative enzymes play an important role. SOD, POD, and CAT are among the three most important anti-oxidative enzymes [5,49]. The accumulation of ROS and MDA was dramatically reduced in the *MsHDZ23*-OE lines compared with WT, implying that the oxidative damage was substantially alleviated in the OE lines. Conversely, the *hb7* mutant showed the opposite trend (Figure 4C,E,F, Figure 6C,E,F, and Figure S4A–C). Correspondingly, three representative anti-oxidative enzymes were significantly induced by *MsHDZ23* overexpression. This suggests that the *MsHDZ23*-OE lines have more effective ROS scavenging systems, which is consistent with the enhanced stress tolerance phenotype of OE lines (Figure 4G–I, Figure 6G–I and Figure S4D–F).

The overexpression of *MsHDZ23* led to an enhanced tolerance to drought stress, which was partly attributed to the lower loss rate of water from leaves (Figure 8B). The slower water loss rate caused the *MsHDZ23*-OE lines to maintain a relatively higher water content during drought stress (Figure 8B). As a result, the leaf wilting of the OE lines was less severe than that of WT, and the majority of leaves maintained a green color and normal turgor (Figure 8A). The decreased leaf evaporation of the *MsHDZ23*-OE lines might be attributed to the reduced stomatal aperture, as the stomata play a crucial role in controlling water transpiration from leaves. It is well documented that ABA acts as a pivotal player in regulating stomatal closure under abiotic stresses [4]. However, the decreased sensitivity of the *MsHDZ23*-OE lines to ABA resulted in decreased stomatal pore size and ultimately improved water retention, although the underlying mechanisms are still unclear.

As shown in the phenotypic data, the *MsHDZ23*-OE lines showed significant tolerance to NaHCO_3_ at the germination and mature seedling stages (Figure 5A and Figure 6A). In the absence of NaHCO_3_, the total chlorophyll content of WT plants was slightly higher than that of transgenic lines. However, upon supplementation with 150 mM NaHCO_3_, the WT plants exhibited a greater reduction in total chlorophyll contents than the *MsHDZ23*-OE lines (Figure 6B). These results suggest that the overexpression of *MsHDZ23* reduces chlorophyll degradation and enhances tolerance to alkaline stress. It is well established that alkaline stress in soil is mainly caused by a high concentration of NaHCO_3_ [50]. A previous study suggests that under alkaline stress, plants may activate much more complicated responsive mechanisms in comparison with plants under other stresses [51]. This may be caused by the multiple toxicities of alkali stress, such as high pH, HCO_3_^−^, CO_3_^2−^, and concomitant Na^+^ in the NaHCO_3_ solution [52,53]. While the precise function of MsHDZ23 in regulating alkaline stress remains unknown, further investigation is warranted to elucidate the underlying mechanisms.

HD-ZIP TFs are usually involved in the development of plant organs, and their absence or overexpression can lead to developmental defects in plants, including vascular [54], epidermal [55], and root hair [56] defects. HD-ZIP also affects plant stress tolerance by regulating organ development [57]. However, in the case of MsHDZ23, it did not exhibit any abnormal growth characteristics aside from leaf shape regulation under normal conditions (Figure S2). From this perspective, *MsHDZ23* holds great potential as an excellent candidate in the genetic improvement of Miscanthus abiotic stress tolerance because it positively regulates plant stress tolerance without exerting any harmful influence on plant growth and productivity.

## 4. Materials and Methods

### 4.1. The Identification of HD-ZIP Genes in M. sinensis

Genome sequences of *M. sinensis* were downloaded from Phytozome (http://www.phytozome.net/) (accessed on 1 November 2019), and HD-ZIP protein sequences of *Arabidopsis* were downloaded from the Arabidopsis Information Resource (TAIR) (https://www.arabidopsis.org/index.jsp) (accessed on 3 November 2019). The hidden Markov model (HMM) profile of HD (PF00046) and ZIP (PF02183) was downloaded from the Pfam database (http://pfam.sanger.ac.uk/) (accessed on 5 November 2019). Local BLASTP was performed using HD-ZIP protein sequences of *Arabidopsis* and HMM profile as queries against the proteome sequences of *M. sinensis* with an e-value cut-off at 1 × 10^−5^. The candidate sequences were filtered via an online conserved domain (CD) search at NCBI to confirm the HD and ZIP domains. For genes with several alternative splicing variants, only the longest sequence was retained. The closest *MsHDZ* orthologs to *Arabidopsis* were predicted via BLASTP searching against the *Arabidopsis* proteome sequences (V11.0) (https://www.arabidopsis.org/) (accessed on 21 November 2019).

### 4.2. Phylogenetic Analysis

The neighbor-joining (NJ) tree was constructed with MEGA software (V 11) [58] using the full-length sequences of HD-ZIP proteins of *M. sinensis* and *Arabidopsis*. The bootstrap analysis was carried out with 1000 replicates. Only support values higher than 50% were shown on the clades. The other parameters were adopted as default.

### 4.3. Plant Materials and Abiotic Stress Treatment

The *M. sinensis* plants were clonally propagated and transplanted into potting soil with comparable sizes. The soil was mainly composed of coconut bran, vermiculite, perlite, humus, and peat (3:1:2:2:12, *w/w*). After being grown for 2 months, the plants were irrigated with salt (0.3 M NaCl and 0.3 M Na_2_SO_4_), alkali (0.315 M Na_2_CO_3_ and 0.315 M NaHCO_3_), and mixed salt–alkali (0.15 M NaCl, 0.15 M Na_2_SO_4_, 0.15 M Na_2_CO_3_, and 0.15 M NaHCO_3_) solutions, respectively. For RNA-seq, the top three fully expanded leaves were collected at 0 and 6 h of each stress treatment. In the case of drought, the plants were grown for 2 months and then subjected to short-term (5 days) or long-term (10 days) drought conditions before being harvested for analysis.The transcriptome analysis was performed at BGI Tech Co., Ltd. (Beijing, China). Each experiment was carried out with three replicates.

### 4.4. Plasmid Construction and Transformation of Arabidopsis

To create an *MsHDZ23* OE construct, the coding region of *MsHDZ23* was amplified via PCR and fused with six copies of the MYC tag at the *N*-terminus under the control of CaMV35S promoter in the modified PBI121-MYC vector with *nptII* (a kanamycin resistance gene) [59]. To investigate the expression pattern of *MsHDZ23*, a 2500 bp genomic DNA sequence from the promoter region of *MsHDZ23* was inserted into the DX2181 vector with *nptII* to drive the *GUS* (β-glucuronidase) reporter gene. The constructs were transformed into *Arabidopsis* Columbia-0 (Col-0) or *hb7* mutant (cs875449) using the Agrobacterium-mediated transformation method [60]. The primers used in this study are listed in Supplementary Table S2. Positive transgenic lines were screened on 1/2 MS plates containing the kanamycin (50 mg mL^−1^). Two homozygous *MsHDZ23*-OE lines or *MsHDZ23*-complemented *hb7* lines were selected for phenotypic analysis under abiotic stress treatments.

### 4.5. Histochemical GUS Staining

The GUS expression patterns of *Pro_MsHDZ23_::GUS* seedlings grown on 1/2 MS medium with different stress were analyzed. The GUS staining solution contained 100 mM sodium phosphate buffer (pH 7.0), 0.5 mg mL^−1^ 5-bromo-4-chloro-3-indolyl β-D-glucuronic acid, 0.1% Triton, and 0.5 mM each of potassium ferricyanide and ferrocyanide. Samples were vacuum infiltrated for 15 to 30 min and incubated at 37 °C for 16 to 24 h. After staining, plant tissues were then fixed in 4% formaldehyde, 50% ethanol, and 5% acetic acid, dehydrated in an ethanol series [61]. Observation was conducted with a light microscope (stemi 508; Zeiss; Suzhou, China).

### 4.6. Subcellular Localization

The full-length sequence (without the termination code) of *MsHDZ23* was amplified and integrated into the pEarleyGate101 via the Gateway LR recombination reaction (Invitrogen, Waltham, MA, USA). The recombinant plasmid and empty vector were separately transformed into agrobacterium EHA105, then transiently expressed in tobacco leaves. The GFP fluorescence was detected using a confocal microscope (TCSsp5Ⅱ03040101, Leica; Wetzlar, Germany). Staining with 4,6-diamidino-2-phenylindole (DAPI) was performed before observation.

### 4.7. Transactivation and Transcriptional Activation Analysis

For the transactivation assay, different truncations of *MsHDZ23* were individually amplified and separately inserted into the pGBKT7 (Clontech; Dalian, China). Each recombinant construct and the empty vector were transformed into yeast AH109. Transformants were sequentially cultivated on SD/-Trp and SD/-Trp-His medium supplemented with 1 mM 3-amino-1,2,4-triazole (3-AT). The growth of yeast colonies and blue colors manifested by β-galactosidase activity against X-α-gal were observed after cultivation at 30 °C for 3 d [62].

Activation assays were carried out in protoplasts prepared from 4-week-old *Arabidopsis* seedlings grown under short-day conditions [63]. The DNA BD from GAL4 was used, and the GAL4 BD-MsHDZ23 fusion proteins could bind to the GAL4 DNA-binding sites of the GUS reporter. The known activator protein MYB3 and suppression protein MYB221 were used as a positive control. The GUS reporter containing four upstream GAL4 DNA-binding sites (GAL4 [4X]-D1-3[4X]-GUS), as well as the luciferase reporter, were cotransformed with GAL4 BD-OsGRF6 into *Arabidopsis* protoplasts. The GUS activity was quantified as described [64]. A plasmid carrying the luciferase gene under the control of the 35S promoter was used as an internal control to normalize the data for variations in the experiment [65].

### 4.8. Seedling Growth Assays

Seed germination assays were performed under various ABA (0.5 and 1.0 μM), NaHCO_3_ (7 mM), Mannitol (100 and 200 mM), and NaCl (50, 100, and 150 mM) treatments. The germination rates were calculated after cultivation for 5 d.

For root elongation assays, seedlings were vertically cultivated on 1/2 MS medium for 3 d, then moved to fresh 1/2 MS medium to grow vertically under ABA (0.5 and 1.0 μM) or NaCl (50, 100, and 150 mM) treatments. The length of primary root was measured after 7 d.

### 4.9. Evaluation of Drought, Salt, and Alkali Tolerance

For drought tolerance assay, seedlings were grown in soil under normal conditions for three weeks. Afterwards, water supply was withheld for 12 d. After re-watering for 3 d, the survival rate was calculated. Leaves were excised and air-dried for 6 h to measure the water loss rate, which was calculated as the proportion of weight loss at each designated time point to the initial weight. To evaluate the salt stress tolerance, first, the seedlings were grown in soil for three weeks, and then, they were adequately irrigated with a NaCl solution (200 mM) at 4 d intervals three times. To study the alkali stress tolerance, the seedlings were grown in the soil for three weeks and were then adequately irrigated with a NaHCO_3_ solution (150 mM) every 3 d for 12 d.

### 4.10. Physiological Measurements and Histochemical Assays

Leaf samples for physiological measurements and histochemical staining were collected from the above drought, salt, and alkali stress assays. The content of MDA was quantified using the previously described method [66]. The proline content was measured using the detection kit (Jiancheng BioEngineering Co., Nanjing, China). For measurement of chlorophyll content, 1 g of fresh leaf samples from genotypes were quickly homogenized in 0.5 mL of 100% acetone followed by addition of 1 mL of 80% acetone to determine the chlorophyll contents according to the method described by Cao et al. [44]. The absorbance values of chlorophyll a and chlorophyll b were, respectively, measured at 663 and 645 nm using an ultraviolet spectro-photometer (UV-2550, Shimadzu, Tokyo, Japan). The enzymatic activities of catalase (CAT), peroxidase (POD), and superoxide (SOD) were measured using commercial detection kits (Jiancheng BioEngineering Co., China).

The in situ accumulation of hydrogen peroxide (H_2_O_2_) and superoxide (O_2_^•−^) in leaves was determined via reactions against 1 mg mL^−1^ 3,3′-diaminobenzidine (DAB) (pH 3.8) and 0.1 mg mL^−1^ nitroblue tetrazolium (NBT) (pH 7.5) solution for 16 h. Following incubation, leaf chlorophyll was removed by soaking in 95% ethanol for 2 h to facilitate visualization. The appearance of brown or blue spots indicated the accumulation of H_2_O_2_ or O_2_^•−^, respectively.

### 4.11. Statistical Analysis

All experiments were carried out with three replicates, and data were expressed as mean ± standard deviation (SD). The significance of differences between different genotypes was examined with one-way analysis of variance (ANOVA) using Duncan’s multiple range test with SPSS software (v18.0). Significant difference was assumed at *p* ≤ 0.05.

## 5. Conclusions

In this study, we functionally characterized *MsHDZ23,* a *Miscanthus* HD-ZIP TF, in the plant abiotic stress response. MsHDZ23 acts as a positive regulator of the plant abiotic stress response in transgenic Arabidopsis. *MsHDZ23* is a nuclear-localized transcription activator. The overexpression of *MsHDZ23* led to enhanced salt, alkali, and drought stress tolerance by stimulating anti-oxidative enzymes and ROS scavenging capability. The expression *of MsHDZ23* was induced by ABA, suggesting that it possibly participate in the plant stress response via an ABA-dependent pathway. Importantly, the overexpression lines of MsHDZ23 showed no detrimental effects on plant growth or development under different stress conditions, indicating the potential of MsHDZ23 as a valuable candidate for improving abiotic stress tolerance in Miscanthus without compromising plant growth and productivity.

## Data Availability

All the data in this study are included in this published article.

## Supplementary Materials

The following supporting information can be downloaded at: https://www.mdpi.com/article/10.3390/ijms25063253/s1.
