# Supplementary material for: MsHDZ23, a Novel Miscanthus HD-ZIP Transcription Factor, Participates in Tolerance to Multiple Abiotic Stresses
Source: Int J Mol Sci. 2024 Mar 13;25(6):3253. doi: 10.3390/ijms25063253 (PMC10970430; doi:10.3390/ijms25063253)
Supplement: Supplementary file 1 [file ijms-25-03253-s001.zip › Supplementary Figures.pdf]

Supplementary files

Figure S1

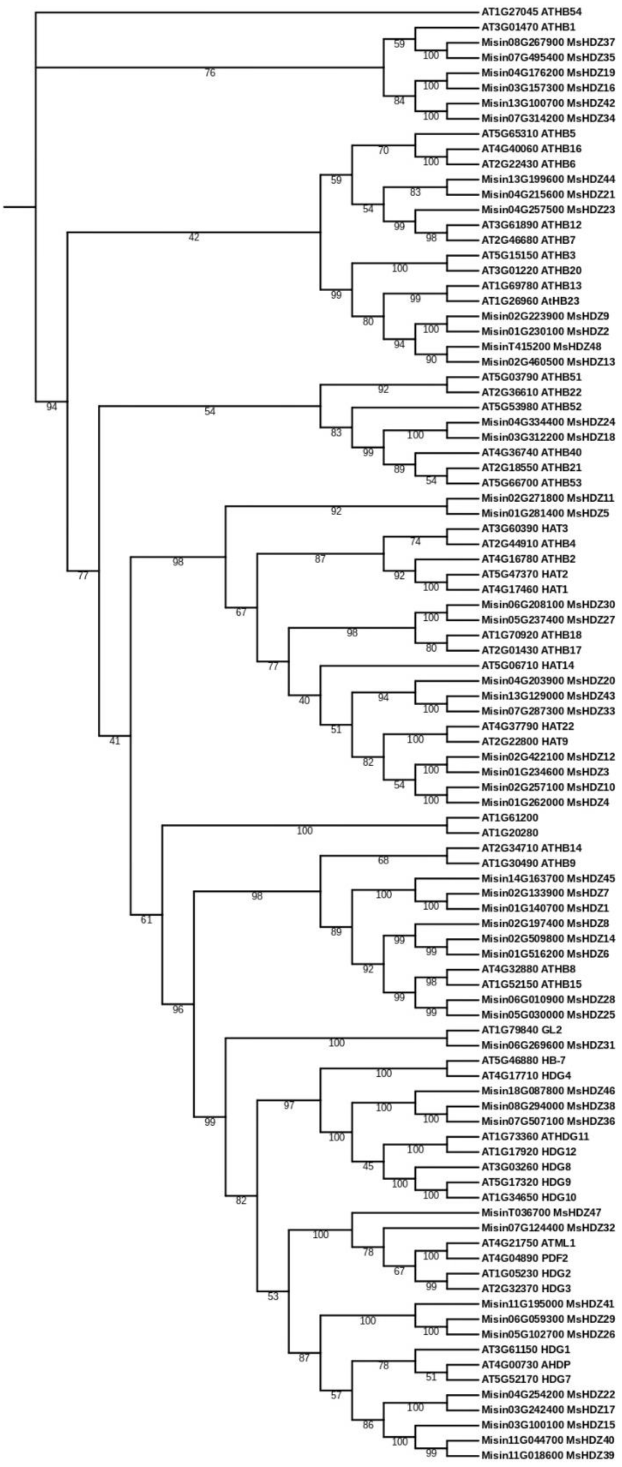

Figure S1. Phylogenetic analysis of HD-ZIP proteins in *M. sinensis* and Arabidopsis. The neighbor-joining (NJ) tree was constructed with MEGA software (Version 11, Mega Limited, Auckland, New Zealand) using the full-length sequences of HD-ZIP

proteins in M. sinensis and Arabidopsis. The bootstrap analysis was carried out with 1000 replicates.

Figure S2

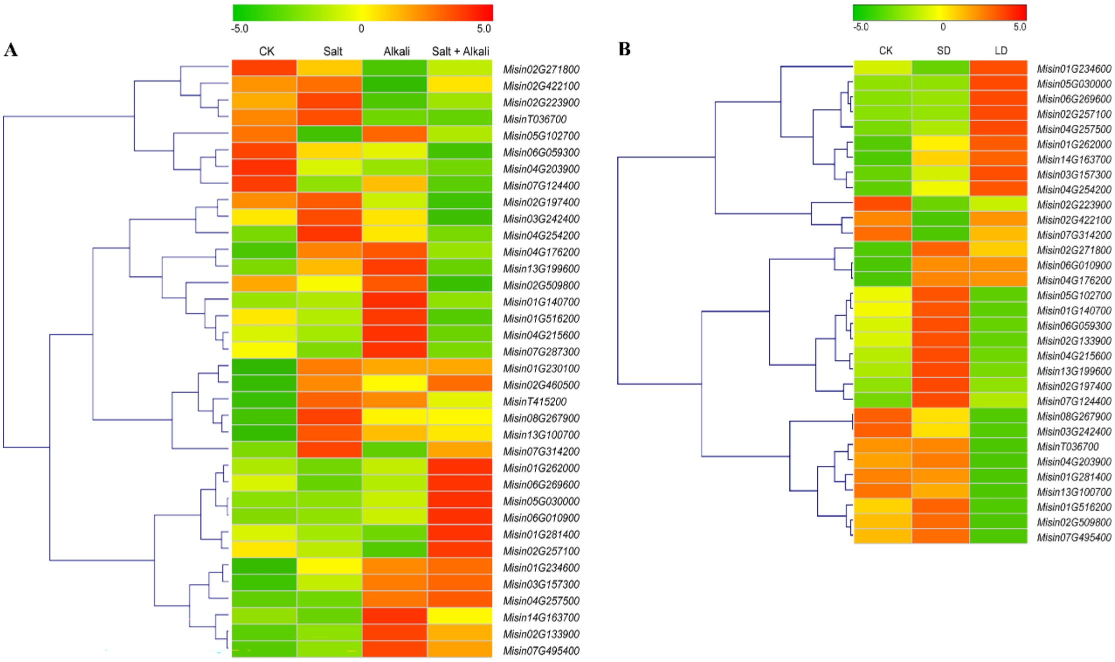

Figure S2. Expression profiling of *MsHD-ZIP* genes under abiotic stresses. (A) Expression profiling of *MsHD-ZIP* genes under salt, alkali, and mixed salt-alkali stresses. The transcriptome sequencing was performed using Miscanthus leaves subjected to salt (0.3 M NaCl and 0.3 M Na_2SO_4), alkali (0.315 M Na_2CO_3 and 0.315 M $NaHCO_3$), and mixed salt-alkali (0.15 M NaCl, 0.15 M Na_2SO_4, 0.15 M Na_2CO_3, and 0.15 M $NaHCO_3$) treatments for 6 h. (B) Expression profiling of *MsHD-ZIP* genes under drought stresses. For drought, after being grown for 2 months, the plants grow for 1 day (short drought) or 5 days (long drought) after the soil completely drought. Each experiment was carried out with three replicates.

Figure S3

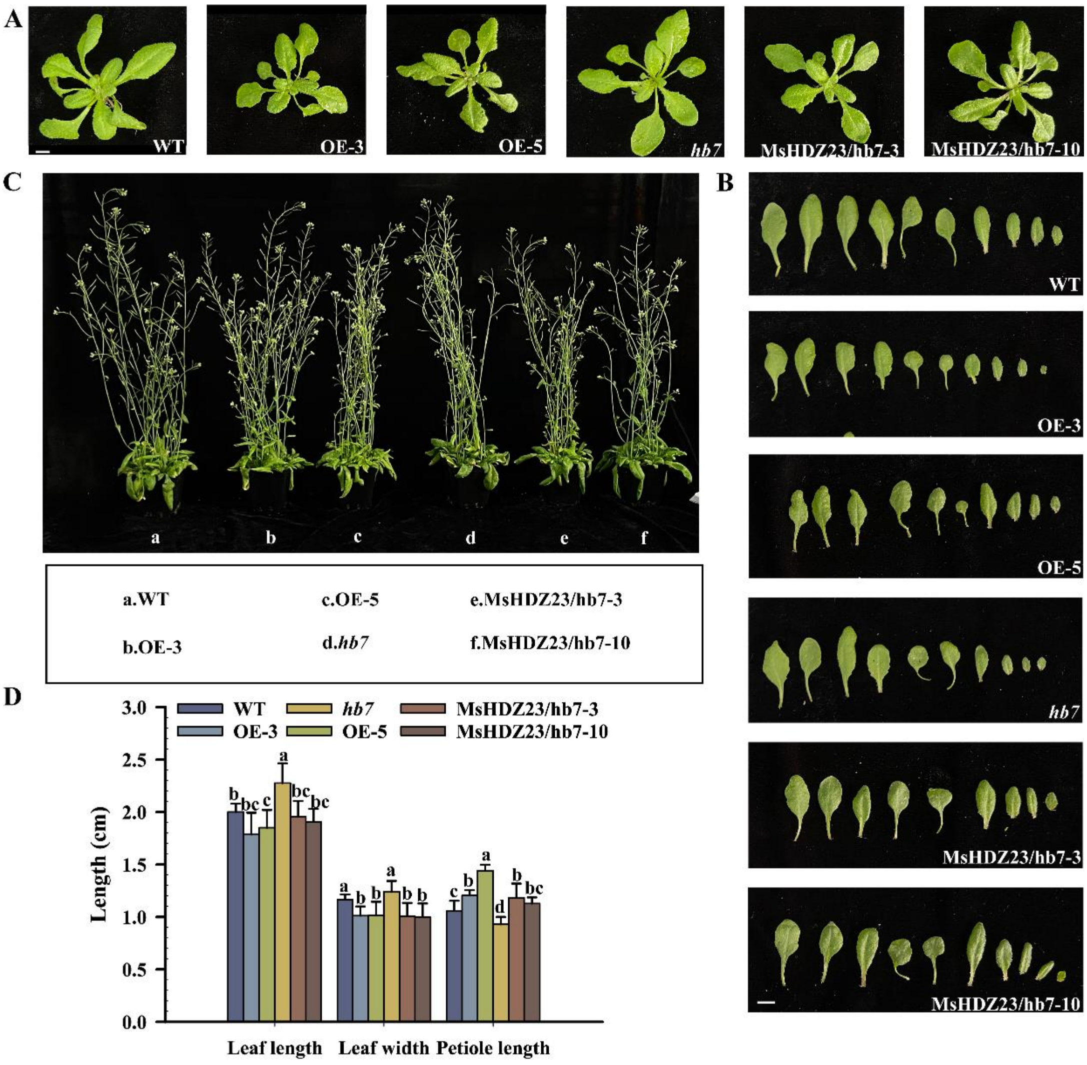

Figure S3. Growth characteristics of *MsHDZ23* transgenesis lines. (A) Five-week-old *MsHDZ23* transgenesis lines, *hb7* mutant and WT plants under long-day (LD) conditions. The diameter of rosette leaves was slightly smaller in the *MsHDZ23*-OE lines compared to the WT. The scale bar is 0.5 cm. (B) Detached rosette leaves of five-week-old *MsHDZ23* transgenesis lines, *hb7* mutant and WT plants under LD conditions. The scale bar is 1 cm. (C) Phenotype of seven-week-old *MsHDZ23* transgenesis lines, *hb7* mutant and WT plants under LD conditions. (D) Quantification of rosette leaves of five-week-old *MsHDZ23* transgenesis lines, *hb7* mutant and WT plants. Data represent mean of 30 leaves (5th and 6th leaves) for each genotype. The different letters represent significant differences between lines ($P \leqslant 0.05$). Error bars indicate the standard deviation.

Figure S4

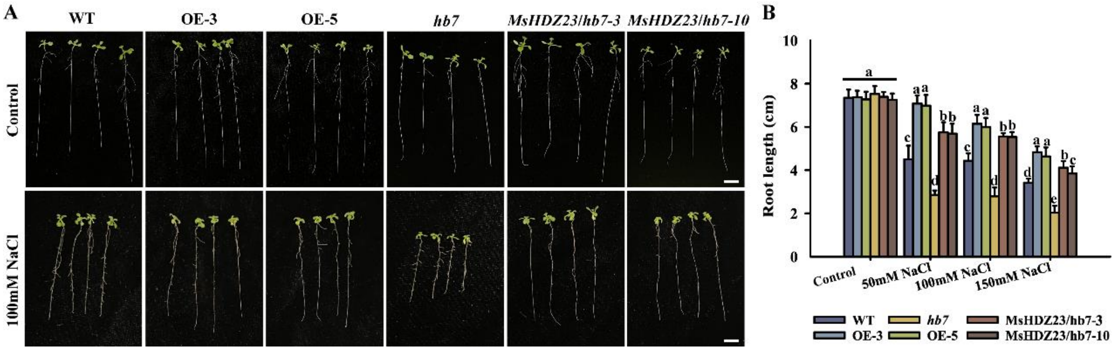

Figure S4. *MsHDZ23* overexpression promotes root growth under NaCl stress. (A) Root elongation of *MsHDZ23* transgenesis lines, *hb7* mutant and WT plants under NaCl stress. Seedlings were vertically grown on 1/2 MS supplemented with different concentrations of NaCl (0, and 100 mM) for 5 d. The scale bar is 1 cm. (B) Quantification of primary root length under different concentrations NaCl treatment (0, 50, 100, and 150 mM) for 5 d. Data are mean of three replicates with 20 plants for each genotype. Error bars indicate the standard deviation. The different letters represent significant differences between lines ($P \leqslant 0.05$).

Figure S5

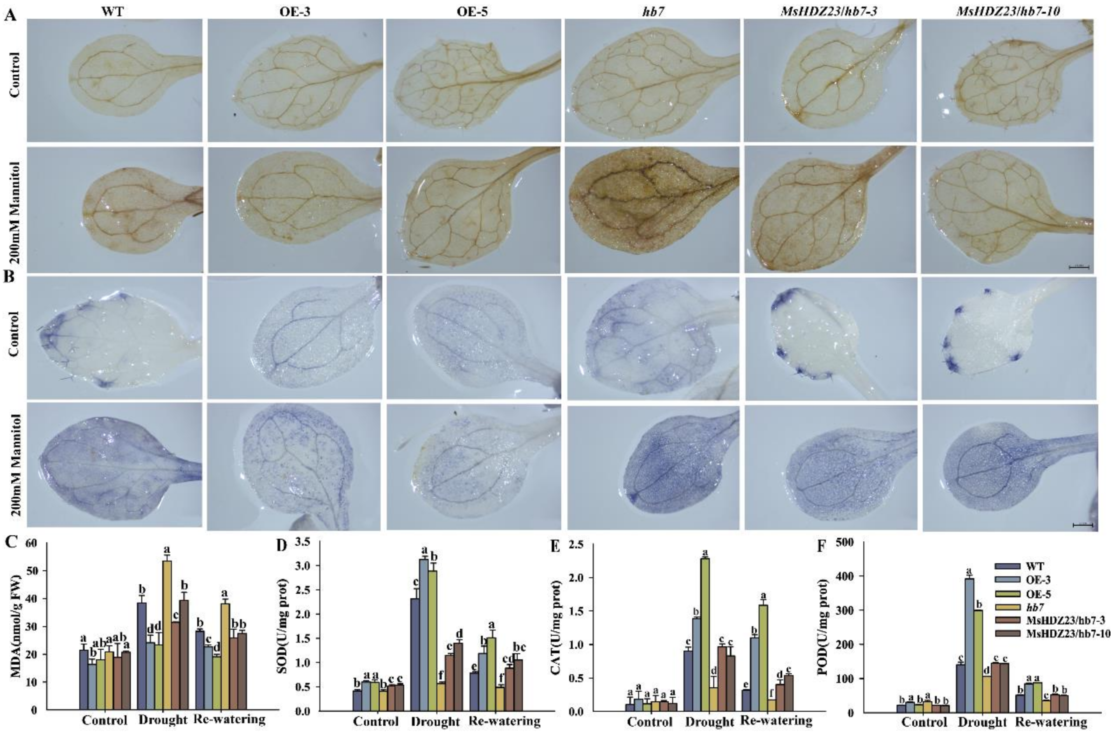

Figure S5. *MsHDZ23* overexpression alleviates ROS accumulation in transgenic Arabidopsis under drought stress. (A, B) Histochemical assays to detect the accumulation of H_2O_2 and $O^{\bullet 2-}$ by DAB (A) and NBT (B) staining under control and mannitol conditions. Seedlings were grown on 1/2 MS supplemented with 0 or 200 mM mannitol concentrations. The scale bar is 0.5 mm. (C- F) Quantification of MDA content and anti-oxidant enzyme activities. The MDA (C) level and enzymatic activities of SOD (D), POD (E), and CAT (F) were measured after drought and re-watering treatment. Values are averages of three independent measurements. Error bars indicate the standard deviation. The different letters represent significant differences between lines ($P \leqslant 0.05$).
